# Correction: An economic analysis of the health-related benefits associated with bicycle infrastructure investment in three Canadian cities

**DOI:** 10.1371/journal.pone.0251031

**Published:** 2021-04-27

**Authors:** David G. T. Whitehurst, Danielle N. DeVries, Daniel Fuller, Meghan Winters

There are errors in the in-text citations for the Supporting Information tables. SM1 refers to S1 Table, SM2 refers to S2 Table, and SM3 refers to S3 Table.

In the Methods section, the heading “Health economic assessment tool” should be written as “Health Economic Assessment Tool”.

The Fig 2 caption is formatted incorrectly. Please see the complete, correct Fig 2 caption here:

**Fig 2:**
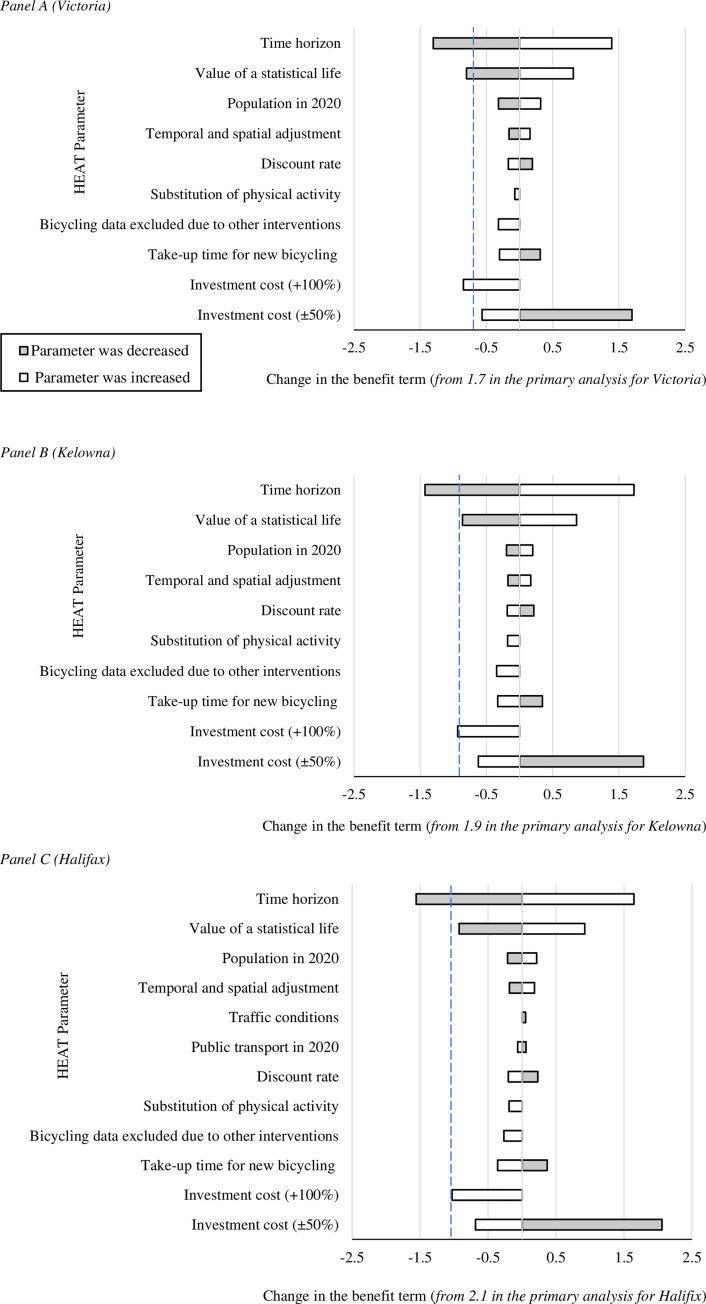
**Findings from the univariate sensitivity analyses for the moderate change (2% increase) scenario in Victoria (Panel A), Kelowna (Panel B) and Halifax (Panel C), where variation in inputs led to a change in the respective benefit:cost ratio. Details of the changes made to the input parameters are provided in S2 Table.** A ‘change’ in a ratio estimate is defined as an absolute difference for the benefit term equal to or greater than 0.05. The results of all sensitivity analyses (to one decimal place) are reported in S3 Table. In each panel, the dashed line indicates the point where the benefit:cost ratio is 1:1.

The S3 Table caption is formatted incorrectly. Please see the complete, correct S3 Table caption here:

**S3 Table. Benefit:cost ratios and the change in the benefit term (when compared with the respective primary analysis) for the 32 univariate sensitivity analyses, by study city.** For comparison, the benefit:cost ratios in the primary analyses for Victoria, Kelowna and Halifax were 1.7:1, 1.9:1 and 2.1:1, respectively.

**Table:** 

Parameter	Victoria		Kelowna		Halifax	
	benefit:cost ratio	*change* in benefit term	benefit:cost ratio	*change* in benefit term	benefit:cost ratio	*change* in benefit term
Time horizon– 50% increase	3.1:1	1.4	3.6:1	1.7	3.7:1	1.6
Time horizon– 50% decrease	0.4:1	-1.3	0.4:1	-1.4	0.5:1	-1.6
Population in 2020–5% increase	2.3:1	0.3	2.3:1	0.2	2.5:1	0.2
Population in 2020–5% decrease	1.1:1	-0.3	1.5:1	-0.2	1.6:1	-0.2
Public transport in 2020–20% increase	1.7:1	<│0.05│	1.9:1	<│0.05│	2.0:1	-0.1
Public transport in 2020–20% decrease	1.7:1	<│0.05│	1.9:1	<│0.05│	2.1:1	0.1
Bicycling data excluded due to other interventions–increase to 20%	1.4:1	-0.3	1.5:1	-0.3	1.8:1	-0.3
Temporal and spatial adjustment–increase to 10%	1.9:1	0.2	2.0:1	0.2	2.2:1	0.2
Temporal and spatial adjustment–decrease to -10%	1.5:1	-0.2	1.7:1	-0.2	1.9:1	-0.2
Take-up time for new bicycling– 50% increase	1.4:1	-0.3	1.5:1	-0.3	1.7:1	-0.4
Take-up time for new bicycling– 50% decrease	2.0:1	0.3	2.2:1	0.3	2.4:1	0.4
New trips–increase to 10%	1.7:1	<│0.05│	1.9:1	<│0.05│	2.1:1	<│0.05│
Bicycling for transportation–increase to 100%	1.7:1	<│0.05│	1.9:1	<│0.05│	2.1:1	<│0.05│
Bicycling for transportation–decrease to 90%	1.7:1	<│0.05│	1.9:1	<│0.05│	2.1:1	<│0.05│
Bicycling in traffic– 20% increase	1.7:1	<│0.05│	1.9:1	<│0.05│	2.1:1	<│0.05│
Bicycling in traffic– 20% decrease	1.7:1	<│0.05│	1.9:1	<│0.05│	2.1:1	<│0.05│
Traffic conditions–increase one category	1.7:1	<│0.05│	1.9:1	<│0.05│	2.0:1	<│0.05│
Traffic conditions–decrease one category	1.7:1	<│0.05│	1.9:1	<│0.05│	2.1:1	0.1
Change in crash risk–increase to 10%	1.7:1	<│0.05│	1.9:1	<│0.05│	2.0:1	<│0.05│
Change in crash risk–decrease to -10%	1.7:1	<│0.05│	1.9:1	<│0.05│	2.1:1	<│0.05│
Substitution of physical activity–increase to 10%	1.6:1	-0.1	1.7:1	-0.2	1.9:1	-0.2
Investment cost– 100% increase	0.8:1	-0.8	0.9:1	-0.9	1.0:1	-1.0
Investment cost– 50% increase	1.1:1	-0.6	1.2:1	-0.6	1.4:1	-0.7
Investment cost– 50% decrease	3.4:1	1.7	3.7:1	1.9	4.1:1	2.1
Carbon values for 2016 & 2025–20% increase	1.7:1	<│0.05│	1.9:1	<│0.05│	2.1:1	<│0.05│
Carbon values for 2016 & 2025–20% decrease	1.7:1	<│0.05│	1.9: 1	<│0.05│	2.0:1	<│0.05│
Discount rate–increase to 3%	1.5:1	-0.2	1.7:1	-0.2	1.9:1	-0.2
Discount rate–decrease to 0%	1.9:1	0.2	2.1:1	0.2	2.3:1	0.2
Value of a statistical life– 50% increase	2.5:1	0.8	2.7:1	0.9	3.0:1	0.9
Value of a statistical life– 50% decrease	0.9:1	-0.8	1.0:1	-0.9	1.1:1	-0.9
Bicycling fatality rate– 20% increase	1.7:1	<│0.05│	1.9:1	<│0.05│	2.0:1	<│0.05│
Bicycling fatality rate– 20% decrease	1.7:1	<│0.05│	1.9:1	<│0.05│	2.1:1	<│0.05│

^a^ The benefit:cost ratios reported in S3 Table correspond to the sensitivity analyses described in S2 Table. Different wording is used to differentiate between absolute (e.g., ‘increase to 10%’) and relative (e.g., ‘10% increase’) changes to parameters. Benefit:cost ratios for all analyses–primary and sensitivity–are reported to one decimal place. These rounded estimates were not used in the calculation of the *change* in the benefit term.
