# Supplementary material for: An economic analysis of the health-related benefits associated with bicycle infrastructure investment in three Canadian cities
Source: PLoS One. 2021 Feb 8;16(2):e0246419. doi: 10.1371/journal.pone.0246419 (PMC7870067; doi:10.1371/journal.pone.0246419)
Supplement: S1 Table — (DOCX) [file pone.0246419.s001.docx]

**S1 Table**. Inputs for the parameters not reported in Table 2 of the manuscript^a^.

| Parameter | Input(s) |
| --- | --- |
| Your Assessment |  |
| Active travel modes^b^ | bicycling |
| Geographic scale^b^ | city level  *country – United Kingdom*  *city – Brighton* |
| Comparison & time scale | two cases^b^  *baseline/reference year – 2016*^c^  *comparison year – 2020*^c^  *calculation of impacts (years) – 10*^d^ |
| Impacts^b^ | physical activity, air pollution, crash risk and carbon emissions |
| Motorized modes^b^ | basic categories  *specified modes – driving and public transport* |
| Data Input |  |
| Active modes data – data source (reference case)^c^ | population survey |
| Active modes data – population type (reference case)^d^ | general population |
| Active modes data – age range of population (reference case)^d^ | adult population (20-64 years) |
| Active modes data – data source (comparison case)^c^ | hypothetical scenario |
| Active modes data – population type (comparison case)^d^ | general population |
| Active modes data – age range of population (comparison case)^d^ | adult population (20-64 years) |

^a^ The label of each parameter reflects the wording used in the respective section and subsection of HEAT v4.2 (as of May 2020).

^b^ User is required to enter a response (i.e., there is no default entry in HEAT).

^c^ Parameter has a default entry in HEAT and the default entry was changed. Some default entries are populated based on responses to earlier questions, e.g., the data source for the comparison case is automatically populated with the same response as entered for the reference case data source.

^d^ Parameter has a default entry in HEAT and the default entry was used.
