# Supplementary material for: An economic analysis of the health-related benefits associated with bicycle infrastructure investment in three Canadian cities
Source: PLoS One. 2021 Feb 8;16(2):e0246419. doi: 10.1371/journal.pone.0246419 (PMC7870067; doi:10.1371/journal.pone.0246419)
Supplement: S2 Table — (DOCX) [file pone.0246419.s002.docx]

**S2 Table**. Parameters and inputs comprising the 32 univariate sensitivity analyses, by study city^a^.

| Parameter | Victoria | Kelowna | Halifax |
| --- | --- | --- | --- |
| Time horizon (years) – 50% increase | 15 | 15 | 15 |
| Time horizon (years) – 50% decrease | 5 | 5 | 5 |
| Population in 2020 (number) – 5% increase | 169,094 | 94,221 | 153,291 |
| Population in 2020 (number) – 5% decrease | 152,990 | 85,247 | 138,691 |
| Public transport in 2020 (min./person/day) – 20% increase | 8.8 | 3.0 | 7.7 |
| Public transport in 2020 (min./person/day) – 20% decrease | 5.8 | 2.0 | 5.1 |
| Bicycling data excluded due to other interventions (%) – increase to 20% | 20 | 20 | 20 |
| Temporal and spatial adjustment (%) – increase to 10% | 10 | 10 | 10 |
| Temporal and spatial adjustment (%) – decrease to -10% | -10 | -10 | -10 |
| Take-up time for new bicycling (years) – 50% increase | 6 | 6 | 6 |
| Take-up time for new bicycling (years) – 50% decrease | 2 | 2 | 2 |
| New trips (%) – increase to 10% | 10 | 10 | 10 |
| Bicycling for transportation (%) – increase to 100% | 100 | 100 | 100 |
| Bicycling for transportation (%) – decrease to 90% | 90 | 90 | 90 |
| Bicycling in traffic (%) – 20% increase | 59 | 80 | 53 |
| Bicycling in traffic (%) – 20% decrease | 39 | 54 | 35 |
| Traffic conditions – increase one category | ‘free flow’ | ‘free flow’ | ‘free flow’ |
| Traffic conditions – decrease one category | ‘heavy’ | ‘heavy’ | ‘heavy’ |
| Change in crash risk (%) – increase to 10% | 10 | 10 | 10 |
| Change in crash risk (%) – decrease to -10% | -10 | -10 | -10 |
| Substitution of physical activity – increase to 10% | 10 | 10 | 10 |
| Investment cost (million $)^b^ – 100% increase | 137.4 | 55.8 | 57.4 |
| Investment cost (million $)^b^ – 50% increase | 103.1 | 41.9 | 43.1 |
| Investment cost (million $)^b^ – 50% decrease | 34.4 | 14.0 | 14.4 |
| Carbon values for 2016 & 2025 ($)^b^ – 20% increase | 51.6 & 63.0 | 51.6 & 63.0 | 51.6 & 63.0 |
| Carbon values for 2016 & 2025 ($)^b^ – 20% decrease | 34.4 & 42.0 | 34.4 & 42.0 | 34.4 & 42.0 |
| Discount rate (%) – increase to 3% | 3 | 3 | 3 |
| Discount rate (%) – decrease to 0% | 0 | 0 | 0 |
| Value of a statistical life (million $)^b^ – 50% increase | 9.75 | 9.75 | 9.75 |
| Value of a statistical life (million $)^b^ – 50% decrease | 3.25 | 3.25 | 3.25 |
| Bicycling fatality rate (fatalities/hundred million km) – 20% increase | 2.676 | 2.676 | 2.676 |
| Bicycling fatality rate (fatalities/hundred million km) – 20% decrease | 1.784 | 1.784 | 1.784 |

^a^ Inputs for the primary, city-level analyses of the moderate scenario are provided across Table 2 and SM1. Different wording is used to differentiate between absolute (e.g., ‘increase to 10%’) and relative (e.g., ‘10% increase’) changes to parameters.

^b^ Monetary inputs are reported in Canadian dollars. HEAT requires inputs (and provides outputs) in euros; we used an exchange rate of $1:€0.65 (January 2016; <https://www.xe.com/>).
