# Supplementary material for: An economic analysis of the health-related benefits associated with bicycle infrastructure investment in three Canadian cities
Source: PLoS One. 2021 Feb 8;16(2):e0246419. doi: 10.1371/journal.pone.0246419 (PMC7870067; doi:10.1371/journal.pone.0246419)
Supplement: S3 Table — For comparison, the benefit:cost ratios in the primary analyses for Victoria, Kelowna and Halifax were 1.7:1, 1.9:1 and 2.1:1, respectively. (DOCX) [file pone.0246419.s003.docx]

**S3 Table**. Benefit:cost ratios and the *change* in the benefit term (when compared with the respective primary analysis) for the 32 univariate sensitivity analyses, by study city. For comparison, the benefit:cost ratios in the primary analyses for Victoria, Kelowna and Halifax were 1.7:1, 1.9:1 and 2.1:1, respectively^a^.

| Parameter | Victoria | |  | Kelowna | |  | Halifax | |
| --- | --- | --- | --- | --- | --- | --- | --- | --- |
|  | benefit:cost ratio | *change* in benefit term |  | benefit:cost ratio | *change* in benefit term |  | benefit:cost ratio | *change* in benefit term |
| Time horizon – 50% increase | 3.1:1 | 1.4 |  | 3.6:1 | 1.7 |  | 3.7:1 | 1.6 |
| Time horizon – 50% decrease | 0.4:1 | -1.3 |  | 0.4:1 | -1.4 |  | 0.5:1 | -1.6 |
| Population in 2020 – 5% increase | 2.3:1 | 0.3 |  | 2.3:1 | 0.2 |  | 2.5:1 | 0.2 |
| Population in 2020 – 5% decrease | 1.1:1 | -0.3 |  | 1.5:1 | -0.2 |  | 1.6:1 | -0.2 |
| Public transport in 2020 – 20% increase | 1.7:1 | <│0.05│ |  | 1.9:1 | <│0.05│ |  | 2.0:1 | -0.1 |
| Public transport in 2020 – 20% decrease | 1.7:1 | <│0.05│ |  | 1.9:1 | <│0.05│ |  | 2.1:1 | 0.1 |
| Bicycling data excluded due to other interventions – increase to 20% | 1.4:1 | -0.3 |  | 1.5:1 | -0.3 |  | 1.8:1 | -0.3 |
| Temporal and spatial adjustment – increase to 10% | 1.9:1 | 0.2 |  | 2.0:1 | 0.2 |  | 2.2:1 | 0.2 |
| Temporal and spatial adjustment – decrease to -10% | 1.5:1 | -0.2 |  | 1.7:1 | -0.2 |  | 1.9:1 | -0.2 |
| Take-up time for new bicycling – 50% increase | 1.4:1 | -0.3 |  | 1.5:1 | -0.3 |  | 1.7:1 | -0.4 |
| Take-up time for new bicycling – 50% decrease | 2.0:1 | 0.3 |  | 2.2:1 | 0.3 |  | 2.4:1 | 0.4 |
| New trips – increase to 10% | 1.7:1 | <│0.05│ |  | 1.9:1 | <│0.05│ |  | 2.1:1 | <│0.05│ |
| Bicycling for transportation – increase to 100% | 1.7:1 | <│0.05│ |  | 1.9:1 | <│0.05│ |  | 2.1:1 | <│0.05│ |
| Bicycling for transportation – decrease to 90% | 1.7:1 | <│0.05│ |  | 1.9:1 | <│0.05│ |  | 2.1:1 | <│0.05│ |
| Bicycling in traffic– 20% increase | 1.7:1 | <│0.05│ |  | 1.9:1 | <│0.05│ |  | 2.1:1 | <│0.05│ |
| Bicycling in traffic – 20% decrease | 1.7:1 | <│0.05│ |  | 1.9:1 | <│0.05│ |  | 2.1:1 | <│0.05│ |
| Traffic conditions – increase one category | 1.7:1 | <│0.05│ |  | 1.9:1 | <│0.05│ |  | 2.0:1 | <│0.05│ |
| Traffic conditions – decrease one category | 1.7:1 | <│0.05│ |  | 1.9:1 | <│0.05│ |  | 2.1:1 | 0.1 |
| Change in crash risk – increase to 10% | 1.7:1 | <│0.05│ |  | 1.9:1 | <│0.05│ |  | 2.0:1 | <│0.05│ |
| Change in crash risk – decrease to -10% | 1.7:1 | <│0.05│ |  | 1.9:1 | <│0.05│ |  | 2.1:1 | <│0.05│ |
| Substitution of physical activity – increase to 10% | 1.6:1 | -0.1 |  | 1.7:1 | -0.2 |  | 1.9:1 | -0.2 |
| Investment cost – 100% increase | 0.8:1 | -0.8 |  | 0.9:1 | -0.9 |  | 1.0:1 | -1.0 |
| Investment cost – 50% increase | 1.1:1 | -0.6 |  | 1.2:1 | -0.6 |  | 1.4:1 | -0.7 |
| Investment cost – 50% decrease | 3.4:1 | 1.7 |  | 3.7:1 | 1.9 |  | 4.1:1 | 2.1 |
| Carbon values for 2016 & 2025 – 20% increase | 1.7:1 | <│0.05│ |  | 1.9:1 | <│0.05│ |  | 2.1:1 | <│0.05│ |
| Carbon values for 2016 & 2025 – 20% decrease | 1.7:1 | <│0.05│ |  | 1.9 :1 | <│0.05│ |  | 2.0:1 | <│0.05│ |
| Discount rate – increase to 3% | 1.5:1 | -0.2 |  | 1.7:1 | -0.2 |  | 1.9:1 | -0.2 |
| Discount rate – decrease to 0% | 1.9:1 | 0.2 |  | 2.1:1 | 0.2 |  | 2.3:1 | 0.2 |
| Value of a statistical life – 50% increase | 2.5:1 | 0.8 |  | 2.7:1 | 0.9 |  | 3.0:1 | 0.9 |
| Value of a statistical life – 50% decrease | 0.9:1 | -0.8 |  | 1.0:1 | -0.9 |  | 1.1:1 | -0.9 |
| Bicycling fatality rate – 20% increase | 1.7:1 | <│0.05│ |  | 1.9:1 | <│0.05│ |  | 2.0:1 | <│0.05│ |
| Bicycling fatality rate – 20% decrease | 1.7:1 | <│0.05│ |  | 1.9:1 | <│0.05│ |  | 2.1:1 | <│0.05│ |

^a^ The benefit:cost ratios reported in SM3 correspond to the sensitivity analyses described in SM2. Different wording is used to differentiate between absolute (e.g., ‘increase to 10%’) and relative (e.g., ‘10% increase’) changes to parameters. Benefit:cost ratios for all analyses – primary and sensitivity – are reported to one decimal place. These rounded estimates were not used in the calculation of the *change* in the benefit term.
