# Supplementary material for: An economic analysis of the health-related benefits associated with bicycle infrastructure investment in three Canadian cities
Source: PLoS One. 2021 Feb 8;16(2):e0246419. doi: 10.1371/journal.pone.0246419 (PMC7870067; doi:10.1371/journal.pone.0246419)
Supplement: Attachment — Submitted filename: Response to reviewers.docx [file pone.0246419.s004.docx]

**General response**: we thank the reviewers for their positive and constructive comments. Below are our point-by-point responses, which highlight any changes that have been made to the manuscript. We hope that the amendments meet with the approval of the reviewers and the Editorial Board and look forward to your response. In this document, all page/line numbers refer to the **clean resubmission**.

------------------------------------------------------------------------------------

**Reviewer #1**: In this paper, an economic analysis has been performed to identify the benefits of infrastructure investment in three Canadian cities. For this paper, World Health Organization’s health economic assessment tool has been used. The topic is interesting, and the paper is well-written. However, several simplified assumptions have been made in the study. For example, the future advancements in technology such as the autonomous vehicles or hybrid vehicles has been ignored. It would be suggested to add a section discussing the shortcomings and limitations of this research and suggestions for future research.

RESPONSE: We thank the reviewer for these positive comments. Text has been added to the discussion section. These additions are not framed as shortcomings and/or limitations of the research because the issues raised by the reviewer are not relevant for HEAT (and our research study is an application of HEAT). Instead, we have addressed the issue of technology advances in our discussion of additional/complementary considerations.

**Pages 17-18, lines 385-390**: “*While this is not a limitation of the current study (the study is an application of HEAT), it is important to recognize that economic assessments are likely to require additional analyses (requiring different methodologies) to complement HEAT, depending on the scope of the decision problem or policy question. For example, cities and stakeholders may be interested in economic impacts of new bicycling infrastructure that are beyond health (e.g., business-related or tourism impacts) or the implications associated with technology advances such as hybrid and autonomous vehicles.*”

**Other comments**

*The title seems to be long. It would be suggested to shorten it. You may also want to remove the second part.*

RESPONSE: In the resubmission, the title of the paper is, “*An economic analysis of the health-related benefits associated with bicycle infrastructure investment in three Canadian cities*”.

*One of the limitations in data adjustments is that it was assumed no temporal or spatial adjustments were needed. It should be noted that transportation-related data may always show temporal or spatial instability. For example, in term of safety, previous studies have shown that crash-related data show temporal instability across different time periods. In addition to safety, other elements studied in this research may show temporal/spatial instability. It would be suggested to add this point as one of the limitations of the study.*

RESPONSE: The reviewer’s comment about temporal or spatial instability is well-received. Indeed, ‘temporal and spatial adjustment’ was one of the parameters varied in the sensitivity analysis. It is not clear to us why the reviewer considers our handling of the ‘temporal and spatial adjustment’ parameter to be a limitation. While our base case assumption was zero (i.e., no adjustment), we looked at a 10% increase and a 10% decrease in the sensitivity analysis. The impact of such variations on the HEAT output (the benefit:cost ratio) is reported for all sensitivity analyses, in graphical form (Figure 2) and in a table (SM3). The transparency of our reporting enables readers to see the relative importance of variation in the different inputs. In our discussion of study limitations, we also highlight the subjective nature of analysis using the HEAT (“*this is a subjective exercise and different analysts are likely to have devised different scenarios*”). No amendments have been made.

**Reviewer #2**: After reading the paper with high interest I think it covers an interesting topic and presents a simple, but straightforward, aim, methodology and results with high concerns for policy makers. However, from my view, the paper presents some gaps that might be revised before being published. Mainly, the literature review is scarce, and introduction and discussion should be broadened to cope with novelties and contributions of the paper regarding the existing literature.

**Main comments**

Introduction. The literature review is scarce, and it should be expanded, highlighting the novelties that the present paper shows and the contributions it is expected to address.

RESPONSE: Details regarding existing literature have been added to the ‘Introduction’ section (see **lines 65-72**), and we have expanded on the description of the contribution of the paper (see **lines 91**-**93**).

**Page 3, lines 65-72**: “*In a 2016 systematic review of economic analyses of active transportation interventions that incorporated physical activity (either implemented, proposed or hypothetical), only two of 29 (7%) studies for which the results were reported as ratios of benefits to costs indicated the respective costs outweighing the respective benefits [6]. Further generalizations in this field of research are hampered by the high level of heterogeneity in all aspects of evaluation (study design, quality and transparency, determination of costs and benefits, etc.). For example, in 25 of the 32 studies included in the systematic review by Brown and colleagues, the measurement of ‘benefit’ extended beyond the impact on physical activity, including a range of health (air pollution and injuries) and non-health (e.g., productivity, travel time, and congestion) factors [6].*”

**Page 4, lines 81-83**: “*This tool has been used to quantify the benefits of active travel (including assessments of current conditions or changes due to investments in active transportation) in European settings [10-12] and, less frequently, in Australia [13] and North America [14,15].*”

**Page 4, lines 91-93**: “*The paper also serves as a comprehensive and transparent account of using HEAT, detailing data sources and assumptions for inputs that may be particularly relevant for researchers and practitioners in the North American context.*”

Discussion. The discussion section does not analyse/discuss the results in depth, neither the implications of the review for policy and management have been tackled. The authors focus on the implications of using HEAT too much, instead of deriving the recommendations for policy making. Actually, they expose just some general statements (lines 290, 344-345). Thus, the authors are encouraged to include the main policy suggestions and recommendations that are derived from the results, applied to the specific cities that they have assessed.

RESPONSE: We are pleased to read that ‘the implications of using HEAT’ comes through as a major focus of the discussion – this was our intention. Our study is an application of HEAT and, as identified in the expanded literature review, the literature on economic analyses in this context suffers from a lack of transparency and quality. We consider the extent of our transparent reporting to be a major strength the paper.

In recognition of the reviewer’s request for further discussion of our findings, the following details have been added to the Discussion section. However, we wish to highlight that our paper is not a policy analysis. For this reason, we believe it to be beyond the scope of the paper to make city-specific policy recommendations. The amendment we have made to the end of the ‘Introduction’ section, in response to the reviewer’s previous comment (**lines** **91**-**93**), provides further clarity regarding the contribution of the paper.

**Page 15, lines 309-310**: “…, *which means that the dollar value of health-related benefits exceeded the cost of planned infrastructure investments in all primary assessments.*”

**Page 16, lines 341-345**: “*For example, the findings in this study were robust to variation in inputs. The cost of infrastructure investments only exceeded (or came close to exceeding) the dollar value of health-related benefits in scenarios where investment costs were doubled, the value of a statistical life was halved, or a significantly shorter time horizon was considered. The identification of plausible ranges of variation for HEAT inputs is an important step in the development of an analysis plan.*”

Discussion. As the authors state, one of the strengths of the work is the collaboration with stakeholders (line 350). However, along the paper there is no references to any stakeholder consultation. Please, in case it does, it should be included in the Methods section (what type of stakeholder has been consulted, how many, when…), and also the main statements derived from this consultation.

RESPONSE: We thank the reviewer for highlighting an area where we can improve the clarity of our paper. As stated on page 4, the IBIMS project was designed in collaboration with stakeholders. The extent of the stakeholder collaboration for the economic analysis project *within* IBIMS focused on the identification of infrastructure costs to support bicycling. We’ve added further information to the Methods section.

**Page 11, lines 217-228**: “*To populate the investment costs parameter, we extracted data from publicly available financial plans and active transportation plans for each of the three study cities [25-28]. Specifically, we sought information about the costs of (i) capital projects for new active transportation infrastructure inclusive of bicycling, sidewalk, and active travel/complete street corridors, and (ii) maintenance of existing infrastructure. All modes of active transportation were included, since some cities had budget lines attached to active corridors rather than bicycle-specific infrastructure. We used 2017 financial plans and captured 2017 capital costs, as well as 2017-2020 operating costs associated with the capital projects. Once initial estimates were derived, we contacted our city partners (planners in the City of Kelowna and Halifax Regional Municipality, and all four municipalities in the Victoria study area) to confirm that our estimates were reasonable approximations of the intended costs. Our conversations with city partners highlighted that costs in budgets were estimates and averages, with the inevitability of variation from year to year. Total cost estimates were discounted to the baseline year (2016), using a discount rate of 1.5% [30].*”

Conclusion. This section is quite general. Please, rewrite it focusing on the main and concrete statements can be derived from the results and the revised discussion.

RESPONSE: The conclusion section in the resubmission now begins with a clear and concise statement about the main finding from the study, mirroring the conclusion in the abstract. Except for a minor wording change (changing ‘the’ to ‘a’ on **line 399**), no other changes have been made.

**Page 18, lines 395-396**: “*Within the assessment framework permitted by HEAT, the dollar value of health-related benefits exceeded the cost of planned infrastructure investments in bicycling in the three study cities (all mid-sized Canadian cities).*”

**Minor comment**

Please, rename the first section as “Introduction” instead of “Background”.

RESPONSE: This change has been made.
